# Supplementary material for: Characterization of the Anti-Viral and Vaccine-Specific CD8+ T Cell Composition upon Treatment with the Cancer Vaccine VSV-GP
Source: Vaccines (Basel). 2024 Aug 1;12(8):867. doi: 10.3390/vaccines12080867 (PMC11359161; doi:10.3390/vaccines12080867)
Supplement: Supplementary file 1 [file vaccines-12-00867-s001.zip › vaccines-3062670-supplementary-revised-v2.pdf]

Characterization of the Anti-Viral and Vaccine-Specific CD8+ T Cell Composition upon Treatment with the Cancer Vaccine VSV-GP

Tamara Hofer [1,2], Lisa Pipperger [1,2,3,4], Sarah Danklmaier [1,2,4], Krishna Das [1,2,5], and Guido Wollmann [1,2,3,4,*]

[1]Institute of Virology, Medical University of Innsbruck, A-6020 Innsbruck, Austria;

tamara.hofer@i-med.ac.at (T.H.); lisa.pipperger@i-med.ac.at (L.P.);

sarah.danklmaier@i-med.ac.at (S.D.); krishna.das@boehringer-ingelheim.com (K.D.)

[2]Christian Doppler Laboratory for Viral Immunotherapy of Cancer, A-6020 Innsbruck, Austria

[3]Department of Internal Medicine V, Haematology & Oncology, Medical University Innsbruck, A-6020 Innsbruck, Austria

[4]Tyrolean Cancer Research Institute, A-6020 Innsbruck, Austria

[5]ViraTherapeutics GmbH, A-6063 Rum, Austria

[*]Correspondence: guido.wollmann@i-med.ac.at

Supplementary information

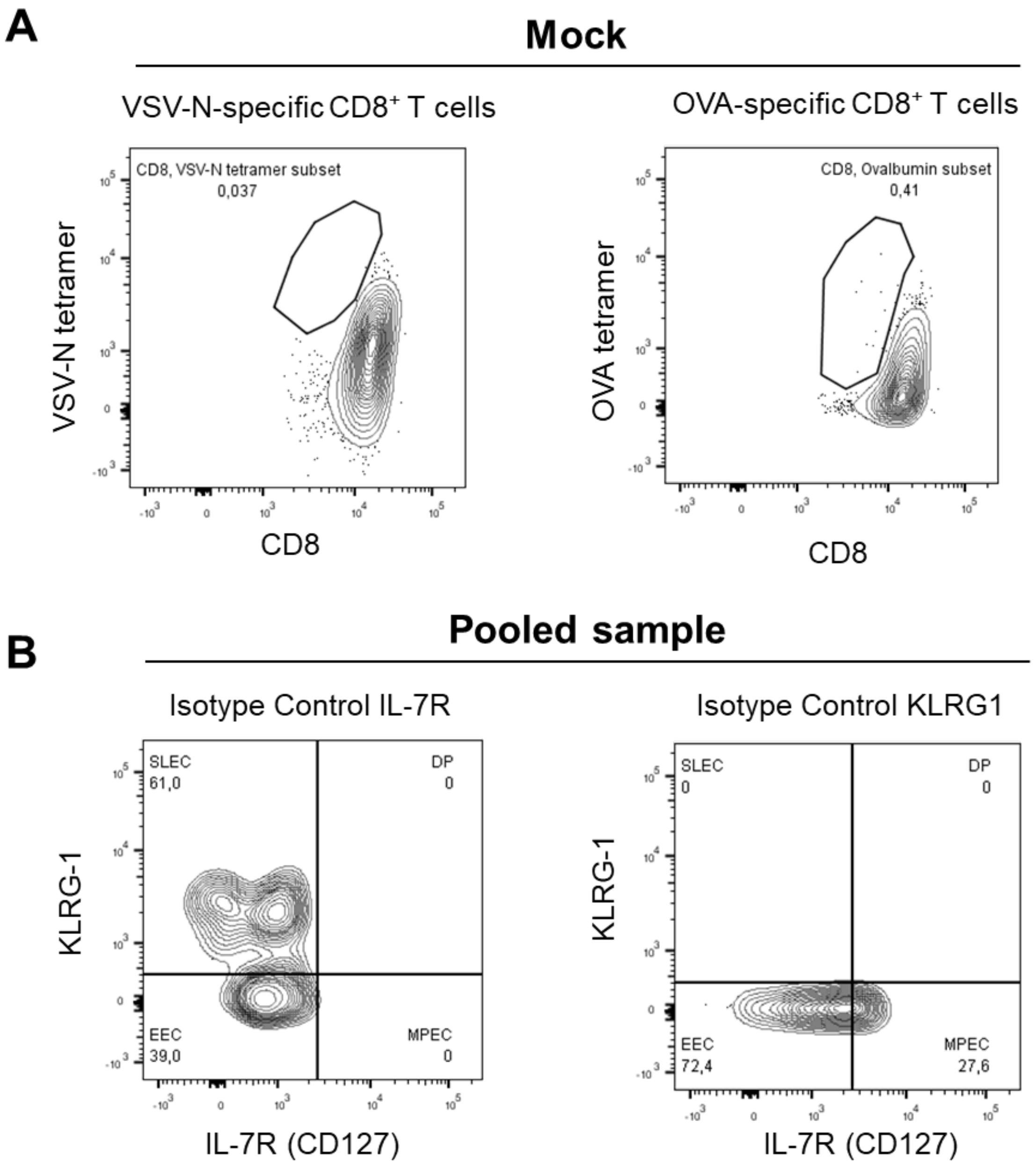

Figure S1: Exemplary contour plots of the detection of VSV-N- and OVA-specific CD8$^+$ T cells and effector subtype isotype controls. (A) Gate placement of VSV-N- and OVA-specific CD8$^+$ T cells in a blood sample of an untreated mouse. **(B)** Identification of early effector cells (EEC, IL-7R$^-$KLRG1$^-$), short-lived effector cells (SLEC, IL-7R$^-$KLRG1$^+$), memory-precursor effector cells (MPEC, IL-7R$^+$KLRG1$^-$) and double-positive (DP, IL-7R$^+$KLRG1$^+$) CD8$^+$ T cells in a pooled blood sample from all treatment groups by using the appropriate isotype control for IL-7R and KLRG1 for correct gate placement.

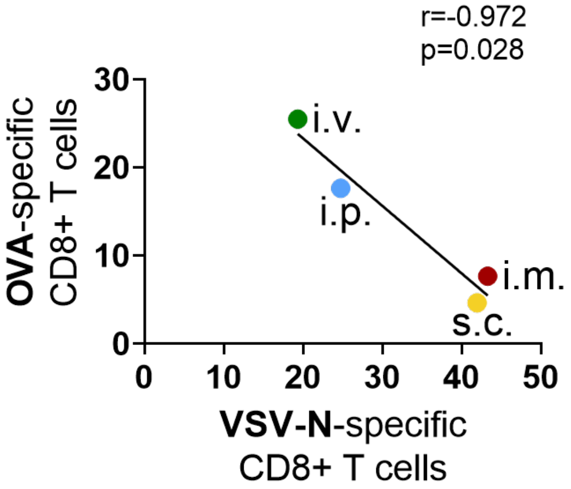

Figure S2: Inverse correlation between OVA-specific and VSV-N-specific CD8$^+$ T cell frequencies concerning different routes of immunization. Six- to eight-week-old female C57BL/6JRj mice were immunized with 10^7 $TCID_{50}$ VSV-GP-OVA intravenously (i.v.), subcutaneously (s.c.), intraperitoneal (i.p) or intramuscularly (i.m.) followed by an equal boost immunization ten days later. Seven days after the boost immunization VSV-N-specific and OVA-specific CD8$^+$ T cell frequencies were assessed. Mean values of different treatment groups (n = 5) were analyzed using the Pearson correlation coefficient. The experiment was performed once. Data shown in this graph has already been shown in Figure 3 and was re-analzyed for statistical comparison to discuss another scientific question.

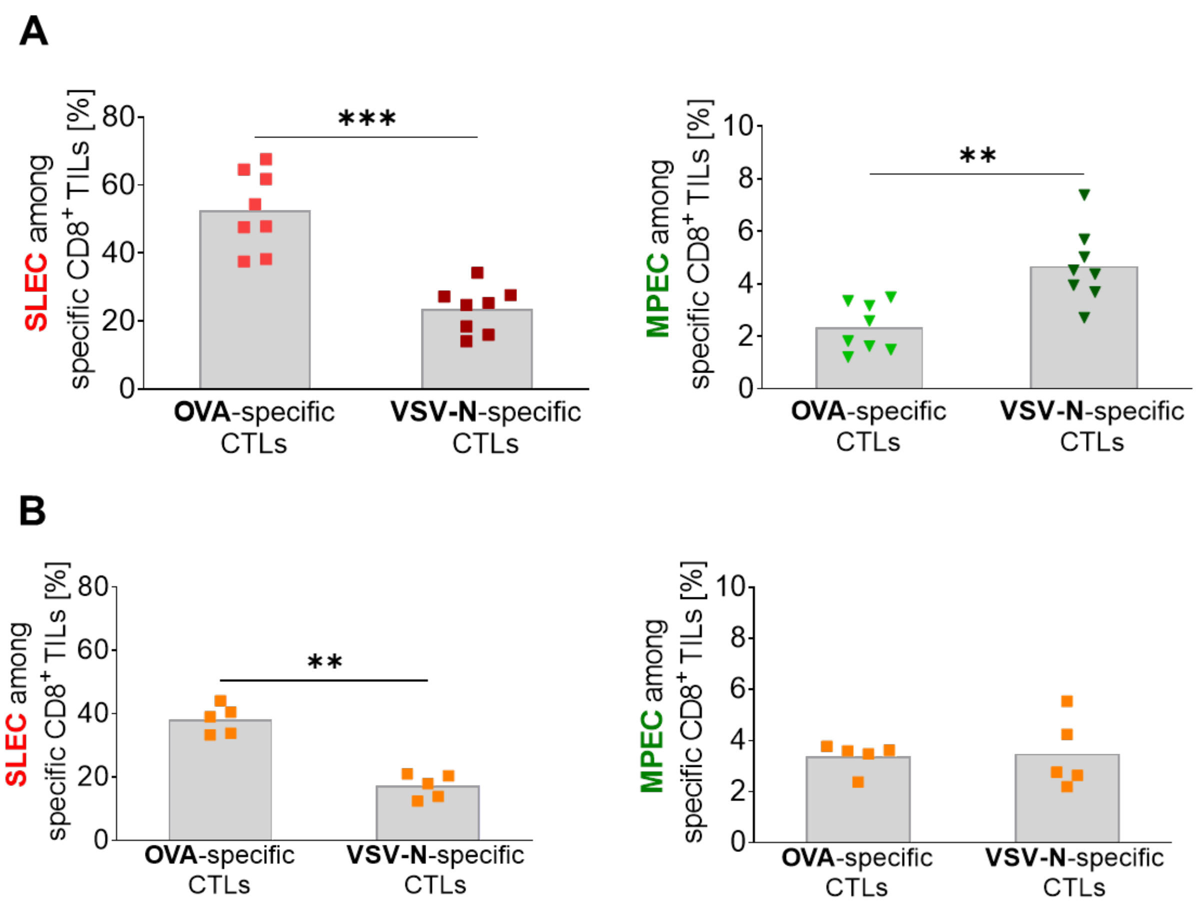

Figure S3: VSV-N- and OVA-specific CD8+ T cells differ in SLEC and MPEC phenotype frequencies in the tumor. B16-OVA tumor cells were subcutaneously (s.c.) implanted into six- to eight-week-old female C57BL/6JRj mice. Mice were treated five days later in a 10-days prime-boost interval with 10^7 $TCID_{50}$ VSV-GP-OVA (V) and 2 nmol KISIMA-OVA (K) in either a homologous (V-V) **(A)** or heterologous (K-V) combination **(B)**. Intratumoral OVA-specific and VSV-N-specific $CD8^+$ T cell SLEC (left) and MPEC (right) frequencies were compared between the different antigen specificities. Mann-Whitney test was performed with n = 8 for **(A)** and n = 5 for **(B)**. Data shown in **(A)** was derived from two independent experiments and data in **(B)** was performed once. Significant differences between treatment groups are indicated with asterisks (** $p \leq 0.01$, *** $p \leq 0.001$). Data shown in this graph has already been shown in Figure 4D, 4E as well as 5D and 5E and was re-analyzed for statistical comparison to discuss another scientific question.

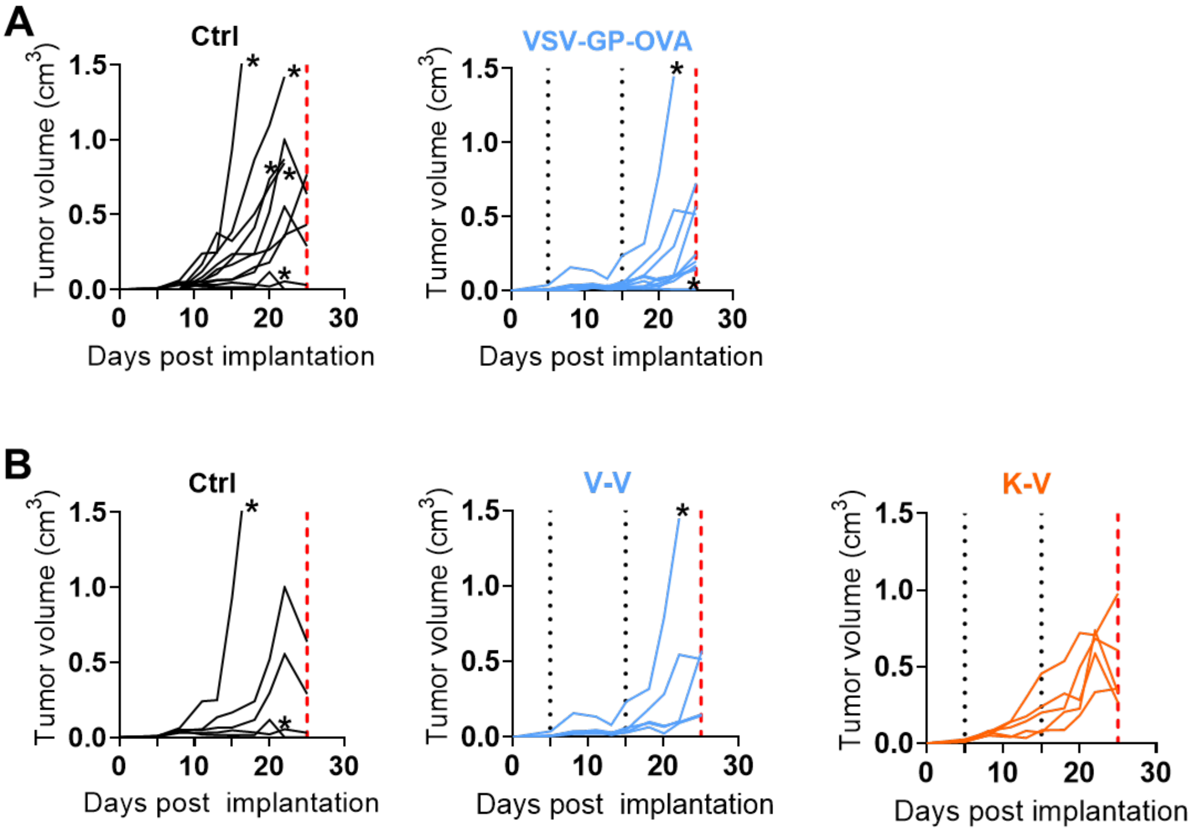

Figure S4: Tumor growth of B16-OVA tumors after homologous or heterologous treatments. B16-OVA tumor cells were subcutaneously (s.c.) implanted into six- to eight-week-old female C57BL/6JRj mice. **(A)** Mice remained either untreated (Ctrl, n = 10) or were treated five days after the implantation in a 10-day prime-boost interval with 10^7 $TCID_{50}$ VSV-GP-OVA (n = 10). The flow cytometry data of blood and tumor tissue of this experiment are shown in Figure 4. **(B)** Five days after B16-OVA tumor cell implantation, mice remained untreated (Ctrl, n = 5) or were treated in a 10-day prime-boost interval with either 10^7 $TCID_{50}$ VSV-GP-OVA (V-V) or with a heterologous combination with 2 nmol KISIMA-OVA (K) and VSV-GP-OVA (10^7 $TCID_{50}$) (K-V, n = 5). Tumor curves of untreated mice (Ctrl, n = 5) and the homologous V-V group (n = 5) are also included in the graphs of section **(A)**. The flow cytometry data of blood and tumor tissue of the experiment in section **(B)** are shown in Figure 5. The dotted lines indicate the time point of vaccination. Ten days post boost, tumors and tumor-draining lymph nodes were harvested for flow cytometric analysis. The red dashed line indicates the harvest time point. Tumors marked with an asterisk were not included in the analysis. Tumors marked with an asterisk were not included in flow cytometric analysis either due to a humane endpoint before 10 post boost or tumor remission.

Table S1: Statistics for Figure 3B and C of OVA- and VSV-N-specific $CD8^+$ T cells in the peripheral blood on day 7, 17, 32, and 60 post VSV-GP-OVA (10^7 $TCID_{50}$) immunization using intramuscular (i.m.), intravenous (i.v.), subcutaneous (s.c.) or intraperitoneal (i.p.) routes. Two-way ANOVA followed by Tukey's multiple comparison test was performed. The experiment was performed once with n = 5 per group.

**OVA-specific $CD8^+$ T cells**								
**Tukey's multiple comparisons test**	**Summary**	**Adjusted P Value**	**Summary**	**Adjusted P Value**	**Summary**	**Adjusted P Value**	**Summary**	**Adjusted P Value**
	7		17		32		60	
**Mock vs. i.m.**	*	0.0313	***	0.0001	ns	0.0906	ns	0.7263
**Mock vs. i.v.**	****	<0.0001	****	<0.0001	****	<0.0001	****	<0.0001
**Mock vs. s.c.**	*	0.03	*	0.046	ns	0.908	ns	0.9903
**Mock vs. i.p.**	****	<0.0001	****	<0.0001	****	<0.0001	*	0.0274
**i.m. vs. i.v.**	ns	0.0637	****	<0.0001	****	<0.0001	**	0.0057
**i.m. vs. s.c.**	ns	>0.9999	ns	0.3517	ns	0.4523	ns	0.9358
**i.m. vs. i.p.**	**	0.0074	****	<0.0001	*	0.0201	ns	0.3949
**i.v. vs. s.c.**	ns	0.0661	****	<0.0001	****	<0.0001	***	0.0004
**i.v. vs. i.p.**	ns	0.9376	****	<0.0001	ns	0.058	ns	0.397
**s.c. vs. i.p.**	**	0.0077	****	<0.0001	****	<0.0001	ns	0.0889
**VSV-N-specific $CD8^+$ T cells**								
**Tukey's multiple comparisons test**	**Summary**	**Adjusted P Value**	**Summary**	**Adjusted P Value**	**Summary**	**Adjusted P Value**	**Summary**	**Adjusted P Value**
	7		17		32		60	
**Mock vs. i.m.**	****	<0.0001	****	<0.0001	****	<0.0001	***	0.0009
**Mock vs. i.v.**	****	<0.0001	****	<0.0001	***	0.0006	ns	0.1096
**Mock vs. s.c.**	****	<0.0001	****	<0.0001	****	<0.0001	ns	0.1311
**Mock vs. i.p.**	****	<0.0001	****	<0.0001	****	<0.0001	ns	0.0548
**i.m. vs. i.v.**	***	0.0001	****	<0.0001	**	0.0015	ns	0.4802
**i.m. vs. s.c.**	ns	0.9995	ns	0.9898	**	0.008	ns	0.4299
**i.m. vs. i.p.**	***	0.0009	****	<0.0001	*	0.0201	ns	0.6655
**i.v. vs. s.c.**	***	0.0002	****	<0.0001	ns	0.985	ns	>0.9999
**i.v. vs. i.p.**	ns	0.976	ns	0.313	ns	0.9202	ns	0.9984
**s.c. vs. i.p.**	**	0.0019	****	<0.0001	ns	0.998	ns	0.9958

Supplementary Table S2: Statistics for Figure 3D, E, F, and G of short-lived effector cells (SLEC) and memory-precursor effector cells (MPEC) among OVA- and VSV-N-specific CD8+ T cells in the blood on day 7, 17, 32 and 60 post VSV-GP-OVA (10^7 $TCID_{50}$) immunization using intramuscular (i.m.), intravenous (i.v.), subcutaneous (s.c.) or intraperitoneal (i.p.) routes are displayed. Two-way ANOVA followed by Tukey's multiple comparison test was performed. The experiment was performed once with n = 5 per group.

SLEC among OVA-specific CD8+ T cells								
**Tukey's multiple comparisons test**	**Summary**	**Adjusted P Value**	**Summary**	**Adjusted P Value**	**Summary**	**Adjusted P Value**	**Summary**	**Adjusted P Value**
	7		17		32		60	
**i.m. vs. i.v.**	**	0.002	*	0.0309	ns	0.4391	ns	0.6099
**i.m. vs. s.c.**	ns	0.9556	ns	0.3624	ns	0.2756	ns	0.6541
**i.m. vs. i.p.**	****	<0.0001	ns	0.9883	ns	0.0692	ns	0.989
**i.v. vs. s.c.**	***	0.0004	***	0.0002	**	0.0079	ns	0.0892
**i.v. vs. i.p.**	ns	0.6871	*	0.0129	***	0.0009	ns	0.4162
**s.c. vs. i.p.**	****	<0.0001	ns	0.5533	ns	0.902	ns	0.8333
**MPEC among OVA-specific CD8+ T cells**								
**Tukey's multiple comparisons test**	**Summary**	**Adjusted P Value**	**Summary**	**Adjusted P Value**	**Summary**	**Adjusted P Value**	**Summary**	**Adjusted P Value**
	7		17		32		60	
**i.m. vs. i.v.**	ns	0.6693	***	0.0002	**	0.0068	ns	0.9291
**i.m. vs. s.c.**	ns	0.9901	ns	0.6053	ns	0.5272	ns	0.9887
**i.m. vs. i.p.**	ns	0.1247	ns	0.7295	ns	0.708	ns	0.9851
**i.v. vs. s.c.**	ns	0.8395	****	<0.0001	****	<0.0001	ns	0.7907
**i.v. vs. i.p.**	ns	0.6906	**	0.0055	ns	0.1089	ns	0.9939
**s.c. vs. i.p.**	ns	0.2253	ns	0.1152	ns	0.0809	ns	0.9084
**SLEC among VSV-N-specific CD8+ T cells**								
**Tukey's multiple comparisons test**	**Summary**	**Adjusted P Value**	**Summary**	**Adjusted P Value**	**Summary**	**Adjusted P Value**	**Summary**	**Adjusted P Value**
	7		17		32		60	
**i.m. vs. i.v.**	****	<0.0001	ns	0.9759	ns	0.4066	ns	0.9216
**i.m. vs. s.c.**	ns	0.0636	ns	0.8925	ns	0.9875	ns	0.9649
**i.m. vs. i.p.**	***	0.0003	ns	0.7576	*	0.0202	ns	0.1979
**i.v. vs. s.c.**	****	<0.0001	ns	0.9908	ns	0.6083	ns	0.6886
**i.v. vs. i.p.**	ns	0.7262	ns	0.9399	ns	0.4884	ns	0.5219
**s.c. vs. i.p.**	****	<0.0001	ns	0.9926	*	0.0475	ns	0.0737

MPEC among VSV-N-specific $CD8^+$ T cells								
**Tukey's multiple comparisons test**	**Summary**	**Adjusted P Value**	**Summary**	**Adjusted P Value**	**Summary**	**Adjusted P Value**	**Summary**	**Adjusted P Value**
	7		17		32		60	
**i.m. vs. i.v.**	ns	0.9961	***	0.0007	*	0.019	*	0.05
**i.m. vs. s.c.**	ns	0.9974	ns	>0.9999	ns	0.9897	ns	0.961
**i.m. vs. i.p.**	ns	0.9681	ns	0.4073	ns	0.4989	ns	>0.9999
**i.v. vs. s.c.**	ns	>0.9999	***	0.0006	*	0.0426	ns	0.1492
**i.v. vs. i.p.**	ns	0.9051	ns	0.0681	ns	0.3837	ns	0.0511
**s.c. vs. i.p.**	ns	0.9149	ns	0.3762	ns	0.6914	ns	0.9631
